# MG132 protects against renal dysfunction by regulating Akt-mediated inflammation in diabetic nephropathy

**DOI:** 10.1038/s41598-018-38425-2

**Published:** 2019-02-14

**Authors:** Wei Zeng, Wei Qi, Jiao Mu, Yi Wei, Li-Ling Yang, Qian Zhang, Qiong Wu, Jian-Ying Tang, Bing Feng

**Affiliations:** Department of Nephrology, Xinqiao Hospital, Third Military Medical University (Army Medical University), Chongqing, People’s Republic of China

## Abstract

Diabetic nephropathy (DN), the leading cause of end-stage renal disease (ESRD). To date, mounting evidence has shown that inflammation may contribute to the pathogenesis of DN. Recent reports have shown that proteasome inhibitors display cytoprotection by reducing the phosphorylation of Akt, a serine/threonine kinase, plays a critical role in cellular survival and metabolism and can crosstalk with inflammation. Therefore, we hypothesized that MG132, specific proteasome inhibitor, could provide renoprotection by suppressing Akt-mediated inflammation in DN. *In vivo*, male Sprague-Dawley rats were divided into normal control group (NC), diabetic nephropathy group (DN), DN model plus MG132 treatment group (MG132), and DN model plus deguelin treatment group (Deguelin)(deguelin, a specific inhibitor of Akt). *In vitro*, a human glomerular mesangial cell lines (HMCs) was exposed to 5.5 mmol/L glucose (CON), 30 mmol/L glucose (HG), 30 mmol/L glucose with 0.5 umol/L MG132 (MG132) and 30 mmol/L glucose with 5 umol/L deguelin (Deguelin). Compared with NC, DN showed a significant increase in the urinary protein excretion rate and inflammatory cytokines, as well as p-Akt. Compared with CON, HMCs co-cultured with HG was notably proliferated, which is in accord with α-smooth muscle actin (α-SMA) expression. These alterations were inhibited by administration of MG132 or deguelin. In conclusion, MG132 significantly inhibits the development of DN by regulating Akt phosphorylation-mediated inflammatory activation.

## Introduction

Diabetic nephropathy (DN) is one of the major causes of microvascular complications of diabetes mellitus (DM) and the leading cause of chronic and end-stage-renal disease worldwide (CKD and ESRD, respectively)^1^. Based on a study in 930 patients with type II diabetes, the Shanghai Diabetic Complications Study reported that the prevalence of microalbuminuria and macroalbuminuria was 22.8% and 3.4%, respectively^2^. Major hallmarks of DN include accumulation of extracellular matrix (ECM) proteins, such as collagens and mesangial expansion in the kidney glomerular and tubular compartments, which contribute to renal failure in diabetes^3–5^. Accumulated data have emphasized the critical role of inflammation in the pathogenesis of DN^6^, which acts through oxidative stress, transcription factors, and inflammatory cytokines. However, the precise mechanisms are unknown.

Akt, a downstream target of activated phosphatidylinositol 3-kinase (PI3K)^7,8^, is activated by mitogens and cytokines. Previous studies have reported the importance of the PI3K/Akt pathway, an important regulator of growth and inflammation, in inflammation-mediated diseases, such as rheumatoid arthritis (RA)^9^ and psoriasis^10^.

In this study, we aimed to determine the effects of high glucose on the development of inflammation and mesangial cell proliferation, as well as mesangial matrix expansion. MG132, specific proteasome inhibitor, prevents damage by inhibiting inflammatory process by regulating Akt and exerts a marked renoprotective effect.

## Material and Methods

### Experimental animal

Male Sprague-Dawley rats (initial weight of 200 to 220 g; average age 8 weeks; Third Military Medical University Animal Experiment Center) were randomly divided into two groups: normal control rats (NC, n = 18) and diabetic nephropathy rats (DN, n = 60). NC rats were fed standard laboratory animal food, while DN rats were fed a high-sugar and high-fat feed (laboratory animal food: Yolk: lard: Sodium cholate: sucrose = 63.5:10:8:0.5:18) for four weeks. And then diabetic rats were subjected to right nephrectomy to hasten the development of kidney disease. After two weeks, DN was induced by intraperitoneal injection with streptozotocin (STZ, 40 mg/kg body weight) dissolved in citrate buffer (pH 4.5, 0.1 M). Blood glucose levels were determined at three and seven days after STZ injection, and only rats with blood glucose levels above 16.7 mmol/L and weakly positive urinary albumin levels were considered as the major indicators of DN, and used in this study. All experimental procedures were carried out in accordance with the recommendations of the Care and Use Committee of the Third Military Medical University. All DN rats (n = 54) were randomly divided into three subgroups, the untreated DN group (DN) and the DN treated with MG132 group (MG132), as well as the DN treated with deguelin group (Deguelin) (deguelin, a specific inhibitor of Akt) which were injected intraperitoneally (i.p.) either with an equal volume of phosphate buffer solution (PBS) alone or with MG132 10 ug/kg (Sigma, US) or with deguelin 4.0 mg/kg (Enzo Life Sciences, Germany) beginning on the day that the DN model was established (week 0). All rats had free access to standard chow and tap water. The 24-h urine samples were collected in metabolic cages at weeks 4, 8 and 12 after treatment began. Rats were sacrificed at the end of weeks 4, 8 and 12 after treatment began, and the left kidneys were harvested, weighed and transversely divided into two pieces, with one piece fixed in 10% formalin for periodic acid-Schiff staining. Other tissues emedullated were frozen in liquid nitrogen for detecting molecular biological expression.

### Measurement of metabolic parameters

All rats were anesthetized and blood samples were drawn from the heart and were centrifuged at 3500 rpm for five minutes. After proper dilution, the supernatant was used for the determination of concentrations of blood glucose (Glu), triglyceride (TG) and total cholesterol (TC) using an enzymatic method (kits from Jiu Qiang biotech company, Beijing, China). The urine protein was determined by chemical method, and the urinary protein excretion rate of 24 h was calculated according to the formula = 24 h total volume of urine (L) × urinary protein levels (mg/L). Urine MCP-1 levels were measured by quantitative sandwich ELISA using a commercial kit according to the manufacturer’s instructions (Biosource, Camarillo, California, USA). The assay was performed in duplicate, and the intensity of the color was measured in an ELISA reader at 450 nm.

### Renal morphologic analysis

Tissue for light microscopy was fixed in 10% formalin and embedded in paraffin. Sections were 4-µm thick and were processed for periodic acid-Schiff staining. Morphologic analyses were performed by an experienced pathologist who was blinded to the source of the tissue. Application of a computer image analysis system for semi-quantitative analysis of the glomerular area: that is, under a low magnification field of vision (×100), thirty glomeruli containing the vascular pole and the urine pole were randomly selected in each slice, and their mean areas were measured and calculated. The glomerular area (GA), defined as the cross-sectional area containing the vascular pole and the urine pole, and the mean areas were measured and calculated. Glomerulosclerosis was defined as index of glomerulosclerosis (IGS). The degree of sclerosis was subjectively graded on a scale of 0 to 4: grade 0, normal; grade 1, affected glomeruli <10%; grade 2, affected glomeruli 10–25%; grade 3, affected glomeruli 25–75%; grade 4, affected glomeruli greater than 75%. IGS was calculated using the following formula: IGS = (1 × N1 + 2 × N2 + 3 × N3 + 4 × N4)/N0 + N1 + N2 + N3 + N4. N is the number of glomeruli in each grade of sclerosis.

### Cell culture

Human glomerular mesangial cells line (HMCs) was kindly provided by Professor Ruan Xiongzhong from Lipds Research Center of Chong Qing Medical University^11^. Transformed HMCs were grown in RPMI 1640 medium (Salt Lake City, UT, USA) supplemented with 5.5 mmol/L glucose and 10% fetal bovine serum (Sijiqing, Hangzhou, China), at 37 °C in a humidified incubator (Heraeus, Germany) with 95% air and 5% CO2. Cells (passages 2–3) grown to sub-confluence were used to complete all the cell based experiments. On this basis, these cells were not passed during the 72 h and the medium was changed every 24 h. HMCs co-culture with 30 mmol/L of glucose (high glucose, HG) is defined as mimicking *in vivo* hyperglucemia, and HMCs were treated with 5.5 mmol/L of glucose would be considered as control. HG with MG132 group was treated with 30 mmol/L of glucose and 0.5 umol/L of MG132, and HG with deguelin group was treated with 30 mmol/L of glucose and 0.1 umol/L of deguelin.

### Cell proliferative rate assay- tetrazolium salt (MTT) colorimetric assay

The cell viability was measured as described previously^12^. Briefly, HMCs were plated on M96-well plates at 1 × 10^4^cells/mL. After the corresponding treatments, we incubated the cells for 4 h with 0.5 mg/mL of MTT (Amersham, LON, UK) and then lysed the cells with dimethylsulfoxide (DMSO). Absorbance was measured at 490 nm in a microplate reader (Sunrise, Austria).

### Quantitative real-time RT-PCR analysis

Total RNA was isolated from the renal tissue using TRIzol extraction (Invitrogen Life Technologies, Shanghai, China) and reverse-transcribed to cDNA using ReverTra AceTM (TOYOBO, Osaka, Japan). Quantitative real-time PCR was performed with primer pairs and probes on a Rotor-gene 6000 (Corbett Life Science, Sydney, Australia). All samples were analyzed in triplicate, and ddH2O served as a no-template control. The relative amount of mRNA was calculated using the comparative Ct (2−ΔΔCt) method. The primer and probe sequences were as follows: (1) NF-κB (forward: 5′-AATTGCCCCGGCAT-3′; reverse: 5′-TCCCGTAACCGCGTA-3′); (2) MCP-1 (forward: 5′-CGCTTCTGGGCCTGTTGTTCC-3′; reverse: 5′-GCCGACTCATTGGGATCATC-3′); (3) TGF-β1 (forward: 5′-ACTGATACGCCTGAGTGGCTGT-3′; reverse: 5′-CTCTGTGGAGCTGAAGCAGTAG-3′); (4) GAPDH (forward: 5′-ACCCATCACCATCTTCCAGGAG-3′; reverse: 5′-GAAGGGGCGGAGATGATGAC-3′).

### Western blot analysis

Tissue samples from the renal tissue were placed in a buffer containing 20 mM Tris-HCl, pH 6.8, 1 mM EDTA, 1% SDS, 1 mM PMSF and 1× protease inhibitor cocktail. The protein was separated on 15% SDS-PAGE and electroblotted onto nitrocellulose (NC) membranes. The membranes were incubated with one of the following antibodies: anti- p65 (1:1000; Cell Signaling Technology, Danvers, MA, USA); anti-p-Akt (Ser^473^,1:1000; Cell Signaling Technology, Danvers, MA, USA); anti-α-SMA(1:1000; Abcam, USA); anti-MCP-1(1:200; Santa Cruz Biotechnology. Santa Cruz, CA, USA); anti-TGF-β1(1:500; Santa Cruz Biotechnology. Santa Cruz, CA, USA);as the primary antibody. HRP-conjugated goat anti-rabbit IgG was used as the secondary antibody (1:1000; Sigma, USA). All membranes were incubated with a monoclonal anti-β-actin antibody (1:2000; Novus, USA). Immunoreactive bands were visualized with the luminescence method (Western Blot Chemiluminescence Reagent Plus, NEN™ Life Science Products Inc.). The band density was normalized to the corresponding density of β-actin at 42 kDa.

### Data analysis

Data were compared among groups using one-way ANOVA, followed by the LSD tests or Mann-Whitney U test. All statistical analyses were performed by the SPSS Statistical Software version 19.0. All values are presented as mean ± S.E.M. and a value of *P* < 0.05 was considered statistically significant. Statistical methods are included in the tables and figures.

## Results

### Metabolic parameters

**Figure 1:**
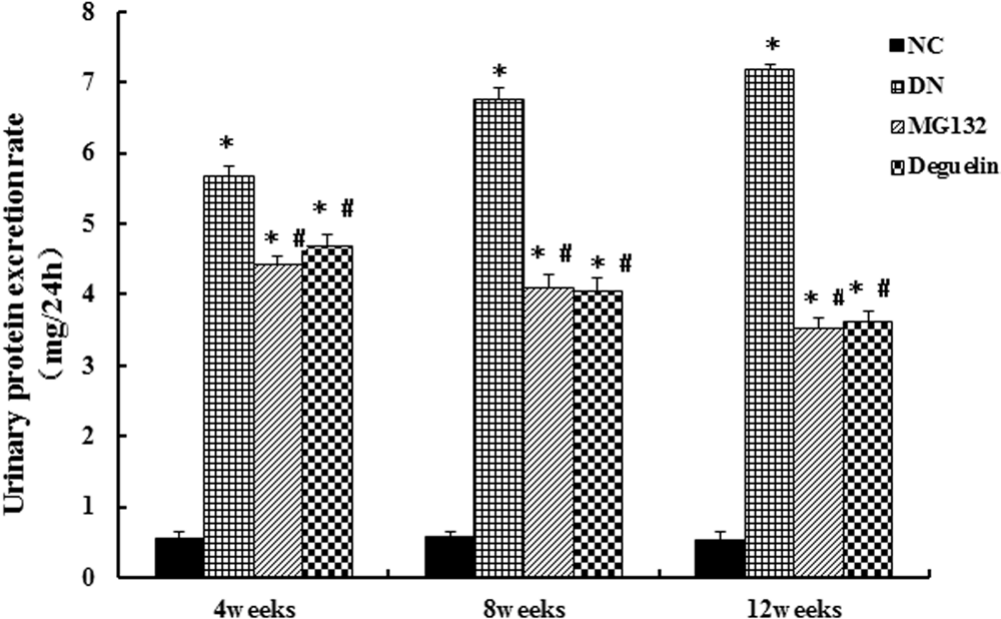
Effect of MG132 on the urinary protein excretion rate in DN rats. In DN rats, both MG132 and deguelin treatment effectively reduced urinary protein excretion for the indicted time. NC: normal control group; DN: diabetic nephropathy group; MG132: diabetic nephropathy plus MG132 treatment group; Deguelin: diabetic nephropathy plus deguelin treatment group. Means ± SEM; N = 6; *P < 0.05 vs. NC; ^**#**^*P* < 0.05 vs. DN.

As shown in Table 1, compared with the NC group, the parameters of the kidney weight/body weight index (KI), Scr, Glu, TG, and TC showed a noticeable increase in DN rats (all *P* < 0.05) at 4, 8, and 12 weeks. Compared to the DN group, treatment with MG132 or deguelin markedly lowed the increase in KI and Glu, especially at 8 and 12 weeks (*P* < 0.05), but had no effect on the metabolism of Scr, TG, and TC. UPER was kept at a low level in NC rats throughout the study period. However, UPER increased progressively with time in DN rats and peaked at the twelfth week (Fig. 1). Treatment with MG132 or deguelin significantly suppressed UPER at 4, 8, and 12 weeks, suggesting that MG132 could effectively prevent the increase in UPER. Therefore, these results indicate that both MG132 and deguelin could markedly prevent renal hypertrophy and renal dysfunction.

**Table 1:** Metabolic parameters in the different groups. The results shown are means ± SEM. NC: normal control group; DN: diabetic nephropathy group; MG132: diabetic nephropathy plus MG132 treatment group; Deguelin: diabetic nephropathy plus deguelin treatment group; BW: body weight; KW: kidney weight; KI: body weight/kidney weight ratio; SCr: serum creatinine; Glu: blood glucose; TG: triglyceride; TC: cholesterol. *P < 0.05 vs. NC; ^#^*P* < 0.05 vs. DN.

Weeks	Groups	N	BW (g)	KW (g)	KI (×10^−3^)	SCr (μmol/L)	Glu (mmol/L)	TG (mmol/L)	TC (mmol/L)
4	NC	6	527.96 ± 2.78	1.26 ± 0.27	2.39 ± 0.75	32 ± 3.15	5.47 ± 0.65	0.57 ± 0.11	1.21 ± 0.04
	DN	6	310.85 ± 1.49*	2.24 ± 0.25*	7.21 ± 0.64*	44 ± 2.60*	24.66 ± 1.54*	1.98 ± 0.42*	2.57 ± 0.15*
	MG132	6	366.15 ± 2.71*	1.66 ± 0.51*	4.53 ± 0.73*	40 ± 2.25*	20.35 ± 2.83*	1.77 ± 0.44*	2.44 ± 0.21*
	Deguelin	6	381.47 ± 1.71*	1.78 ± 0.49*	4.66 ± 0.54*	41 ± 1.93*	20.67 ± 1.84*	1.92 ± 0.37*	2.19 ± 0.30*
8	NC	6	541.80 ± 2.92	1.41 ± 0.19	2.60 ± 0.58	34 ± 2.71	5.66 ± 0.49	0.52 ± 0.09	1.20 ± 0.12
	DN	6	270.66 ± 3.52*	2.99 ± 0.14*	11.05 ± 1.69*	57 ± 3.32*	27.48 ± 3.31*	2.26 ± 0.65*	3.00 ± 0.23*
	MG132	6	398.69 ± 2.80*^#^	1.87 ± 0.42*^#^	4.69 ± 0.97*^#^	46 ± 3.28*	18.48 ± 2.17*^#^	2.00 ± 0.14*	2.61 ± 0.18*
	Deguelin	6	425.1 ± 1.99*^#^	1.87 ± 0.65*^#^	4.40 ± 0.91*^#^	44 ± 1.98*	16.62 ± 2.04*^#^	1.97 ± 0.37*	2.74 ± 0.19*
12	NC	6	547.13 ± 3.31	1.72 ± 0.66	2.88 ± 1.43	34 ± 2.64	5.81 ± 0.63	0.57 ± 0.28	1.22 ± 0.38
	DN	6	248.11 ± 4.48*	3.27 ± 0.51*	13.18 ± 2.79*	64 ± 2.19*	31.59 ± 1.90*	2.31 ± 0.41*	3.18 ± 0.25*
	MG132	6	425.17 ± 2.52*^#^	2.08 ± 0.32*^#^	4.89 ± 1.53*^#^	49 ± 3.33*	12.64 ± 1.34*^#^	2.06 ± 0.06*	2.94 ± 0.46*
	Deguelin	6	440.84 ± 2.22*^#^	2.11 ± 0.35*^#^	4.79 ± 1.08*^#^	47 ± 3.08*	11.95 ± 1.73*^#^	2.01 ± 0.48*	2.99 ± 0.17*

### Effect of MG132 on renal histopathologic changes

**Figure 2:**
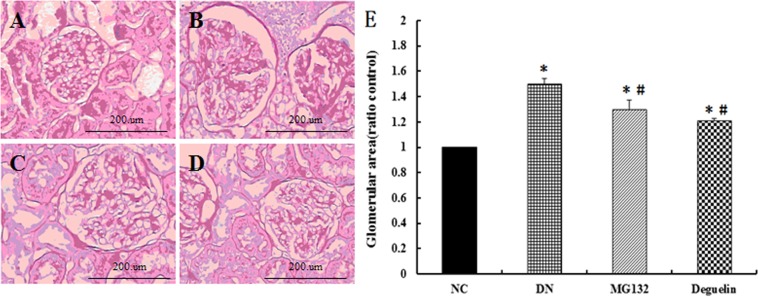
Effect of MG132 on histology in DN rats. In DN rats, both MG132 and deguelin treatment effectively reduced mesangial cell proliferation, and mesangial matrix accumulation for the indicted time. NC: normal control group at the end of 12 weeks (**A**); DN: diabetic nephropathy rats group at the end of 12 weeks (**B**); MG132: diabetic nephropathy plus MG132 treatment group at the end of 12 weeks (**C**); Deguelin: diabetic nephropathy plus deguelin treatment group at the end of 12 weeks (**D**); Glomerular area (ratio NC group) in all rats (**E**). Representative photomicrographs of neutral formaldehyde (10%)-fixed sections stained with PAS are shown. Magnification ×200. Means ± SEM; N = 6; *P < 0.05 vs. NC; ^**#**^*P* < 0.05 vs. DN.

In this study, we found typical glomerular damage in the kidneys of DN rats, including mesangial cell proliferation, mesangial matrix accumulation and expansion(λ), compared with NC (Fig. 2A). Treatment with MG132 and deguelin prevented these changes (Fig. 2C,D). As Fig. 2E shows, for DN, the mean GA was approximately 1.5-fold that of NC at 12 weeks. However, administration of MG132 or deguelin decreased GA by approximately 18% and 20%.

### MG132 suppresses high glucose-induced HMC proliferation

**Figure 3:**
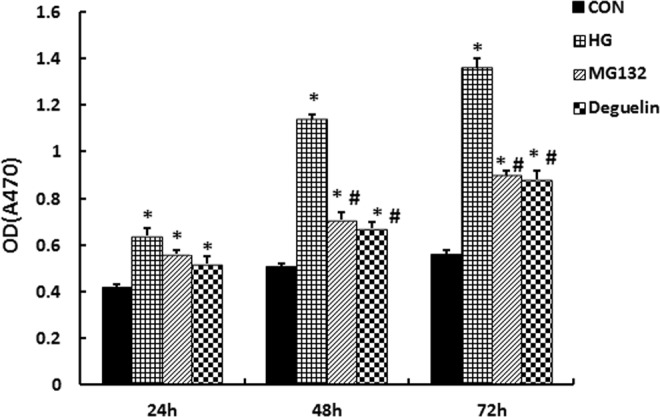
Effect of MG132 on HMCs proliferation. Exposure to 30 mmol/L glucose activated the proliferation of HMCs, manifesting as an increase of the absorbance value of MTT. Both MG132 and deguelin treatment effectively reduced the proliferation of HMCs. CON: normal glucose medium containing 5.5 mmol/L glucose; HG: high glucose containing 30 mmol/L glucose; MG132: high glucose containing 30 mmol/L glucose with MG132; Deguelin: high glucose containing 30 mmol/L glucose with deguelin. *P < 0.05 vs. CON; ^**#**^*P* < 0.05 vs. HG.

To investigate the effect of MG132 on the proliferation of HMCs, HMCs proliferation was detected by the MTT assay. As shown in Fig. 3A, compared with the CON group, high glucose facilitates HMCs proliferation along with the temporal elongation. However, incubation with MG132 or deguelin inhibited high glucose-induced HMCs proliferation.

### MG132 suppresses high glucose-induced expression of α-SMA

**Figure 4:**
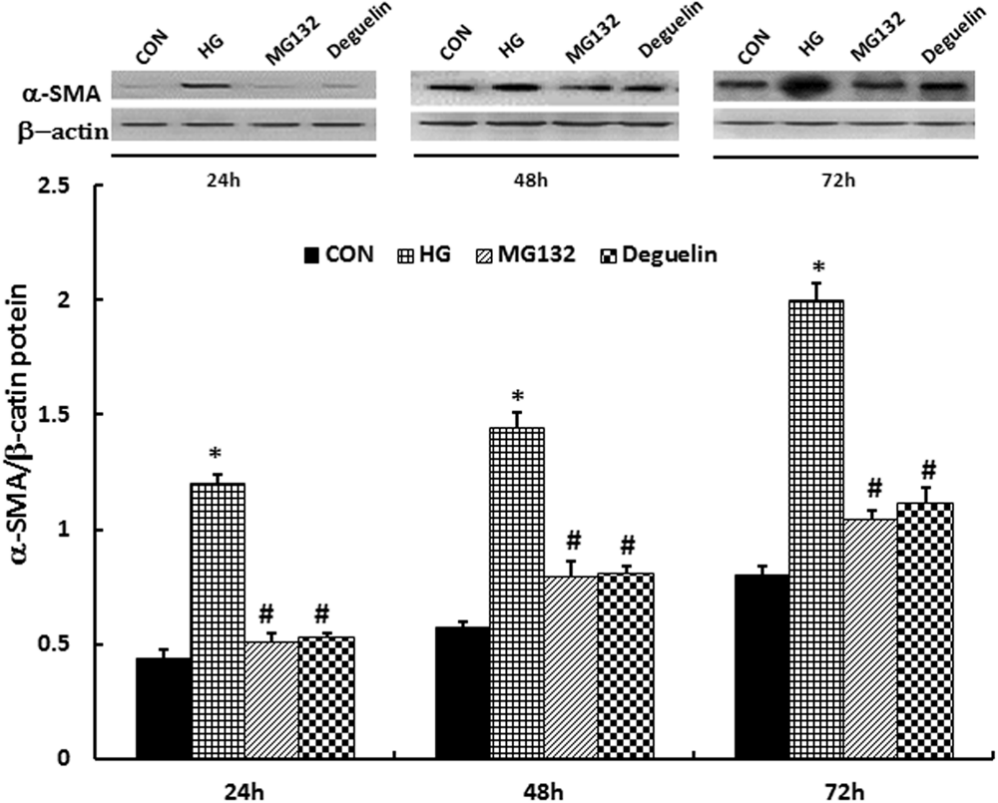
MG132 reversed the high-glucose induced increase of α-SMA. the levels of α-SMA was significantly higher than in CON and were reduced after administration of MG132 and deguelin for the indicted time. α-SMA expression in HMCs was detected by western blotting: HMCs were treated with 5.5 mmol/L (CON) or 30 mmol/L (HG) high glucose for 24 h, 48 h, and 72 h; then, the HG group was treated with MG132 or deguelin. CON: 5.5 mmol/L glucose; HG: 30 mmol/L glucose; MG132: 30 mmol/L glucose with MG132; Deguelin: 30 mmol/L glucose with deguelin; means ± SEM; N = 6; *P < 0.05 vs. CON; ^**#**^*P* < 0.05 vs. HG.

ECM accumulation plays crucial roles in early renal hypertrophy and late glomerular sclerosis in diabetic nephropathy; α-SMA as one of the important indicators of fibrosis; therefore, we evaluated the effect of MG132 on the expression of α-SMA. As shown in Fig. 4, α-SMA was significantly higher in the HG group. After treatment with MG132 or deguelin for 24, 48, and 72 h, α-SMA was significantly decreased at all timing points.

### Effect of MG132 on the renal sclerotic degree

**Figure 5:**
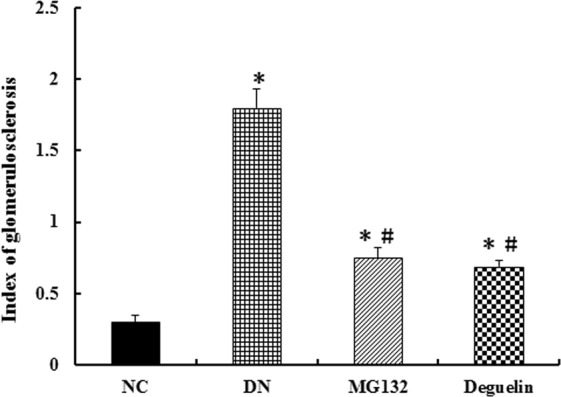
Effect of MG132 on sclerotic degree in DN rats. In DN rats, both MG132 and deguelin treatment effectively reduced the sclerotic degree for the indicted time. NC: normal control group at the end of the study (12 weeks); DN: diabetic nephropathy group at the end of the study (12 weeks); MG132: diabetic nephropathy plus MG132 treatment group at the end of the study (12 weeks); Deguelin: diabetic nephropathy plus deguelin treatment group at the end of the study (12 weeks). Means ± SEM; N = 6; *P < 0.05 vs. NC; ^**#**^*P* < 0.05 vs. DN.

IGS is index for evaluating the sclerotic degree of glomerulosclerosis. As Fig. 5 shows, IGS in the DN group was prominent at 12 weeks, but MG132 and deguelin inhibited the sclerotic degree by approximately 65% and 70%, respectively.

### Effect of MG132 on Akt phosphorylation

**Figure 6:**
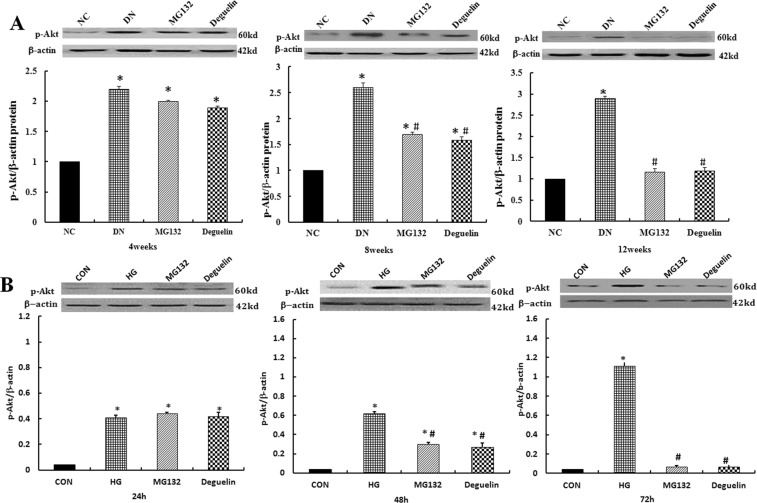
MG132 reversed the high-glucose induced increase of p-Akt(Ser^473^). (**A**) p-Akt(Ser^473^) expression in renal tissue was detected by western blotting: the level of p-Akt(Ser^473^) in the DN group was significantly higher than in the NC group and was reduced after administration of MG132 and deguelin for the indicted time. NC: normal control group; DN: diabetic nephropathy group; MG132: diabetic nephropathy plus MG132 treatment group; Deguelin: diabetic nephropathy plus deguelin treatment group. (**B**) p-Akt(Ser^473^) expression in HMCs was detected by western blotting: HMCs was treated with 5.5 mmol/L (CON) or 30 mmol/L (HG) high glucose for 24 h, 48 h, and 72 h; then, the HG group was treated with MG132 or deguelin. CON: 5.5 mmol/L glucose; HG: 30 mmol/L glucose; MG132: 30 mmol/L glucose with MG132; Deguelin: 30 mmol/L glucose with deguelin; means ± SEM; N = 6; *P < 0.05 vs. NC or CON; ^**#**^*P* < 0.05 vs. DN or HG.

Akt is a well-established protein that regulates cell growth, survival and anti-apoptotic mechanisms. Akt activation is regulated through phosphorylation. Renal tissue western blotting (Fig. 6A) demonstrated that p-Akt(Ser^473^) protein expression was increased in the DN group; however, compared with the DN group (*P* < 0.05), p-Ak(Ser^473^) augmentation in the DN group was partially reversed by MG132. There was no significant difference between the MG132 and deguelin groups, indicating that the proteasome inhibitor MG132 partially reversed the p-Akt(Ser^473^) increase in DN. In addition, similar to the *in vivo* experiment (Fig. 6B), the relative expression of p-Akt(Ser^473^) increased with time in the HG group; the most significant changes were observed after 72 h. After MG132 or deguelin intervention, p-Akt (Ser^473^) expression was significantly decreased. These data suggest that high glucose led to p-Akt(Ser^473^) expression; however, elevated p-Akt(Ser^473^) expression was significantly decreased by the addition of MG132.

### Effect of MG132 on the expression of NF-κB

**Figure 7:**
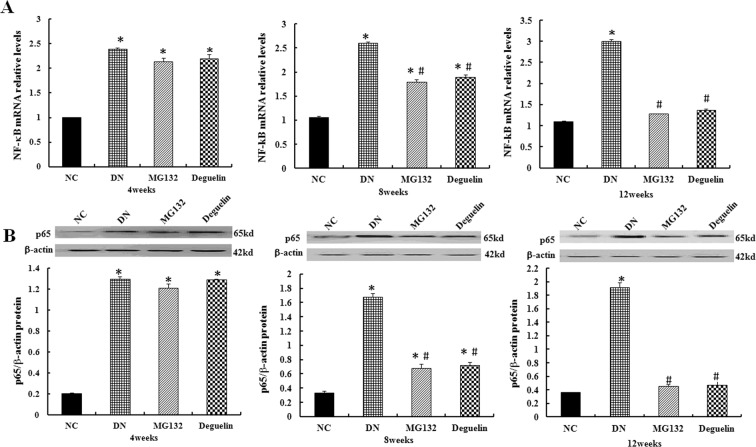
Effect of MG132 on the NF-κB level in DN rats. In DN rats, the relative mRNA level of NF-κB was significantly higher than in NC rats and was reduced after the administration of MG132 and deguelin for the indicted time (**A**). Likewise, the level of p65 was significantly higher than in NC rats and was reduced after administration of MG132 and deguelin for the indicted time (**B**). NC: normal control group; DN: diabetic nephropathy group; MG132: diabetic nephropathy plus MG132 treatment group; Deguelin: diabetic nephropathy plus deguelin treatment group. Means ± SEM; N = 6; *P < 0.05 vs. NC; ^**#**^*P* < 0.05 vs. DN.

NF-κB is a pleiotropic transcription factor that is mainly represented by the p65/p50 heterodimeric complex, which is found in multiple cell types. It regulates the transcription of multiple genes and is involved in the inflammatory response, cell proliferation, and apoptosis^13^. To further elucidate the protective mechanisms of MG132 on the diabetic kidney, we measured the mRNA of NF-κB and protein level of p65. As Fig. 7A shows, significantly elevated of NF-κB expression was detected in the DN group compared to the NC group (*P* < 0.05). Treatment with MG132 reduced the extent of the change. Similarly, deguelin remarkably decreased the expression level of NF-κB after 8 and 12 weeks (*P* < 0.05). We also found that the change trend of NF-KB is consistent with p65 (Fig. 7B); the DN group demonstrated a significant elevation compared with the NC group. However, MG132 and deguelin efficiently inhibited the expression of p65.

### Effect of MG132 on inflammatory cytokine expression

**Figure 8:**
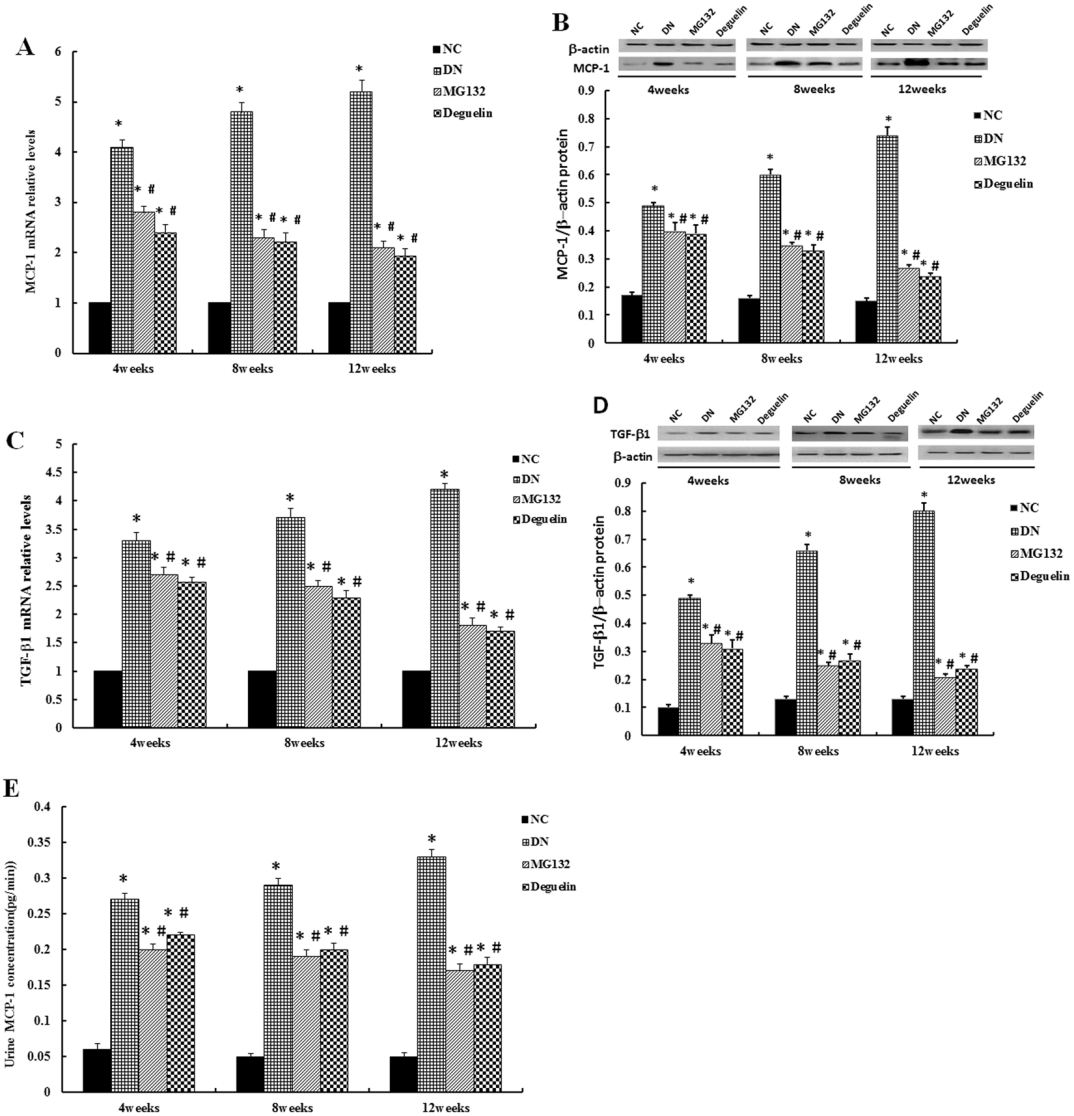
Effect of MG132 on inflammatory cytokine expression in DN rats. MCP-1 mRNA expression was examined by RT-PCR (**A**), and MCP-1 (**B**) protein expression levels were determined using Western blot. In DN rats, the levels of MCP-1 were significantly higher than in NC rats and were reduced after administration of MG132 and deguelin for the indicted time. TGF-β1 mRNA expression was examined by RT-PCR (**C**), and TGF-β1 (**D**) protein expression levels were determined using Western blot. In DN rats, the relative levels of TGF-β1 were significantly higher than in NC rats and were reduced after administration of MG132 and deguelin for the indicted time. In DN rats, the concentration of urine MCP-1 was significantly higher than in NC rats and was reduced after administration of MG132 and deguelin for the indicted time (**E**). NC: normal control group; DN: diabetic nephropathy group; MG132: diabetic nephropathy plus MG132 treatment group; Deguelin: diabetic nephropathy plus deguelin treatment group. Means ± SEM; N = 6; *P < 0.05 vs. NC; ^**#**^*P* < 0.05 vs. DN.

MCP-1, a member of the CC chemokine family of proinflammatory cytokines^14,15^, plays an important role in the propagation of focal inflammation and macrophage infiltration^16^. As Fig. 8A,B shows, the level of MCP-1 was significantly increased compared to the NC group (*P* < 0.05), but MG132 and deguelin effectively suppressed this increase (*P* < 0.05). TGF-β1, another proinflammatory cytokine, is a pivotal mediator of matrix accumulation that results in the development of glomerulosclerosis^17–19^. In this study, we found that the expression level of TGF-β1 was elevated in the DN group compared with the NC group (all *P* < 0.05). However, treatment with MG132 and deguelin decreased the level of TGF-β1 (all *P* < 0.05). Moreover, the urinary MCP-1 concentration was in accordance with the MCP-1 level of the tissues, and the MCP-1concentration was decreased by treatment with MG132 and deguelin (Fig. 8E, *P* < 0.05).

## Discussion

This study demonstrated that the proteasome inhibitor MG132 had a preventative effect on impaired renal function induced by persistent high glucose. Several factors support this concept. First, HMCs co-cultured with high glucose noticeably proliferated, while there was a depressant effect when MG132 was added. MG132 also decreased blood glucose, the urinary protein excretion rates, and glomerulosclerosis in DN rats. Second, high glucose increased the expression of α-SMA and inflammatory transcripts; however, these expression levels were markedly reduced by MG132. Third, p-Akt(Ser^473^) was elevated by hyperglycemia and was significantly attenuated by the administration of MG132. More importantly, the effect of MG132 was in parallel with deguelin, a specific inhibitor of Akt. These results provide the first evidence that MG132 effectively prevents the progression and development of DN. The mechanism might involve the regulation of Akt phosphorylation, which is associated with attenuation of inflammation.

Over the past decades, inflammation, cell hypertrophy, and dedifferentiation further contribute to DN. Despite these advances, currently available therapies are still not fully effective in preventing progression to ESRD suggesting that further molecular mechanisms underlying the pathogenesis of DN is necessary for the improved management of this disease. Recently, alterations of Akt activity have been found in various tissues and cells in experimental and clinical contexts. Akt belongs to a family of serine/threonine protein kinases and is ubiquitously expressed in mammals^20^, regulating cell proliferation, survival, metabolism, migration, and metastasis^21^. In this study, we also found that Akt was activated by hyperglycemia in kidneys, which was evidenced by an increase in p-Akt(Ser^473^). This result is supported by Liu, who demonstrated that the basal level of PI3K/Akt-dependent signalling was increased in diet-induced insulin resistance^22^, which is a key component of multiple metabolic diseases, such as metabolic syndrome, type 2 diabetes mellitus, atherosclerotic heart and brain disorders, fatty liver, Alzheimer’s disease, some types of cancer, and aging^23–31^. Activation of Akt occurs through phosphorylation, which is dependent on the signalling pathways of PI3K and activates mTORC1 through inactivation of tuberous sclerosis complex 2 (TSC2) within the TSC1–TSC2 complex^32^. However, increased activation of mTORC1 triggers a negative feedback loop on the PI3K/Akt pathway, leading to suppression of Akt^33,34^. In this study, we found that HMCs incubated with high glucose demonstrated increased proliferation, which is consistent with the expression of p-Akt(Ser^473^). However, deguelin effectively decreased the level of proliferation. These results suggest that Akt plays a significantly role in the pathology of chronic renal injury.

The most important finding in this study is that we first demonstrated that MG132 has an equivalent effect on alleviating renal deterioration induced by high glucose as deguelin, as evidenced by *in vitro* and *in vivo* studies. *In vivo* research showed that MG132 effectively reduced mesangial cell proliferation, mesangial matrix accumulation, and urine protein excretion for the indicted time in diabetic nephropathy rats. *In vitro* studies also revealed that most mesangial cell phenotypic transformation markers induced by high glucose were suppressed by MG132, including decreased mesangial cell proliferation and the expression of α-SMA. These findings are in line with Sternesjo^35^, who implicated the proteasome in interleukin-1β–mediated suppression of islet function. Interesting, we also found that MG132 supressed the expression of p-Akt(Ser^473^). In particular, Tang^36^ demonstrated that proteasome inhibitors, clasto-lactacystin blactone (LA) or epoxomicin (Epo) reduced p-Akt and activation of autophagy in ARPE-19 cells, possibly through inhibition of PI3K/Akt/mTOR signalling. Therefore, we speculated that MG132, a proteasome inhibitor, would be a drug of practical value for the treatment of diabetic nephropathy through inhibition of the Akt signalling pathway.

Recently, it is believed that DN is one kind of chronic inflammation. Persistent and enhanced inflammation, and finally leads to excessive fibronectin production and extracellular matrix accumulation resulting in acceleration of the pathogenesis of glomerular sclerosis and tubulointerstitial fibrosis. The ubiquitin-proteasome system (UPS) is related to inflammatory signal transmission, such as NF-κB and its downstream signalling cascade. NF-κB is mainly represented by the p65/p50 heterodimeric complex and this complex is retained in the cytoplasm in an inactive form bound to an additional inhibitory subunit – IκBα^37^. During activation, the inhibitory subunit IκBα is rapidly phosphorylated at Ser32 and Ser36 by IKKα/β and subsequently ubiquitinated and degraded by the 26S proteasome complex. Once released, free NF-κB translocates to the nucleus and activates the transcription of various inflammatory gene products. MG132 plays a pivotal role in blocking the degradation of ubiquitinconjugated proteins and permeable strains of yeast by the 26S complex. It inhibits NF-κB activation by reducing the degradation of IκBα. We found that expression of NF-κB and p65 were significantly higher in the DN treated group, as compared with the NC group. In MG132-treated rats the expression of NF-κB and p65 were down-regulated, as compared with DN rats. These results indicated that MG132, inhibited activation of NF-κB. Similar to UPS, Akt appears to require IKK to efficiently stimulate the transactivation domain of the p65 subunit of NF-κB^38^. Deguelin, a specific Akt inhibitor, it suppressed NF-κB, suggesting specificity toward NF-κB. Asha *in vitro* kinase assays showed that deguelin is not a direct inhibitor of IKK, but this agent seems to block the activation of IKK by interfering with upstream regulatory kinases^39^. Other evidence indicated that IKK is a downstream target of Akt. Bhandari provided indirect evidence that renal cortical matrix accumulation in Type 2 DM is, at least in part, attributable to Akt effects^40^. Another new finding of the study is that, deguelin inhibited the high expression of NF-κB and p65 in DN group. In our previous research have shown that renal 26S proteasome activity and concentration, the indicators of UPS, were significantly higher in DN rats than in NC rats at the end of 4, 8 and 12 weeks; these increase reflects the activation of UPS in kidney of DN rats^41^. Therefore, it is reasonable to assume that administration of MG132 and deguelin may constitute a new molecular basis for the inhibition of inflammatory activation in rats with diabetic nephropathy by interruption of activated Akt.

Increasing evidence suggests that inflammation due to proinflammatory cytokines and chemokines secreted by renal cells and macrophages infiltrating the kidney can substantially contribute to DN. In this study, it is interesting to note that there was a significant increase in NF-κB in the DN group compared with the NC group. Furthermore, the results also showed that MCP-1 was significantly elevated in the kidneys of the DN group. Meanwhile, the data in this study demonstrated that UPER was increased in line with urinary MCP-1. More importantly, we found that MG132 not only reduced NF-κB but also reduced the expression of MCP-1 in DN group kidney tissue and decreased urine excretion. NF-κB, the major inflammatory transcription factor that triggers the transcription of several inflammation mediators, such as endothelin-1 (ET-1), VCAM-1, intercellular adhesion molecule-1 (ICAM-1), IL-6, and TNF-α^42^, is expressed in mesangial cells^43^, renal tubule cells, and podocytes in individuals with DM^44^. MCP-1, which is a member of the CC chemokine family of proinflammatory cytokines, plays an important role in the propagation of focal inflammation and macrophage infiltration. Several recent studies have indicated that MCP-1 null mice are protected against DN and blockade of the MCP-1 receptor, C-C chemokine receptor type 2 (CCR-2), using propagermanium-ameliorated diabetic glomerulosclerosis. However, expression of urinary MCP-1 and the secretory volume of UPER were decreased with MG132 administration. Previous studies have shown that urinary excretion of MCP-1 is correlated with diabetic glomerular injury^45^, as well as an increased risk of death and cardiovascular events^46,47^. These results are supported by Banba^45^, whose study indicated that increases in MCP-1 expression and interstitial macrophage infiltration coincide with the development of hyperglycemia and precede a rise in albuminuria in type 1 DN in mice. Bondar^48^ and Wolkow^49^ documented that urinary excretion of proinflammatory factors in patients with DN correlated with the excretion of urine albumin. In a model of STZ-induced type 1 DN, mice genetically deficient in MCP-1 were found to have reduced renal injury compared with wild-type mice with equivalent hyperglycemia. Therefore, MCP-1 plays a critical role in diabetic kidney impairment caused by inflammation, and the proteasome inhibitor MG132 inhibited inflammation and reduced the excretion of urine protein in DN rats. Major hallmarks of DN include the accumulation of ECM proteins, such as collagens (leading to fibrosis), and mesangial expansion (leading to hypertrophy) in the kidney glomerular and tubular compartments, which contribute to renal failure in diabetes. However, the molecular mechanism of this phenomenon has not been established. To verify this hypothesis, we incubated HMCs with high glucose and determined the expression of α-SMA; we found that the level of protein expression was remarkably increased. TGF-β1, the most abundant TGF-β family member isoform, is a pleiotropic cytokine that has been established as a central mediator of kidney inflammation and fibrosis; TGF-β1 is involved in inflammatory responses associated with the NF-κB pathway and binds to latent TGF-β-binding protein (LTBP) and initiates downstream signals^50^. In the present research, we provided evidence that increased expression of TGF-β1 was significantly inhibited by treatment with MG132. These results were supported by the work of Ma^51^, who found that MG132 significantly attenuated hypertension-induced cardiac remodelling and dysfunction via downregulation of TGF-β1. These results were also supported by Sakairi^52^, who confirms rat renal fibroblasts NRK-49F cells and tubular epithelial cells, NRK-52E, were treated with TGF-β in the presence or absence of a proteasome inhibitor, MG132 or lactacystin. Proteasome inhibitors attenuate TGF-β signalling by blocking Smad signal transduction *in vitro*. As mentioned above, MG132 effectively inhibited renal inflammation and fibrosis through attenuation of NF-κB in DN rats. Similarly, administration of deguelin greatly diminished the expression of NF-κB and MCP-1, as well as TGF-β1, suggested that MG132 inhibition of inflammation is in line with deguelin and is associated with NF-κB. It is notable that deguelin alleviates inflammation; whether this is a direct action on NF-κB needs to be investigated. In this study, deguelin suppressed NF-κB activation through a variety of stimuli, suggesting that it must act at a step common to all of these activators.

It is worth noting that MG132 decreased blood glucose compared with DN, which is supported by Zhou^53^, who found that glucose-dependent insulinotropic polypeptide receptor (GIP-R) was rescued by treating isolated islets with the proteasomal inhibitors lactacystin and MG132. After inhibition, the islets were once again capable of increasing the intracellular cAMP levels in response to increase insulin secretion and subsequent effects on glucose metabolism^54–56^. Hofmeister suggested that glucokinase aggregation due to proteasome blocking with MG132, bortezomib, epoxomicin or lactacystin could be detected in MIN6 cells^57^. Similarly, deguelin could also decrease blood glucose, which is related with relieving insulin resistance^58–60^. In recent years, more and more evidences (clinical and animal experiment) suggest DN can’t be prevented by simply lowering blood glucose owing to the “metabolic memory”, supported by Kowluru^61^,who found in diabetic rats, poor glucose control led to hyperglycemia-induced changes in retinal cell apoptotic marker expression, which were sustained for as long as several months following glucose normalization. Not only diabetic nephropathy itself is associated with inflammation, but also we have confirmed that MG132 and deguelin can reduce transcription factor and its expression of inflammatory factors, so as to reduce proteinuria. Whatever the mechanism, these findings indicate that MG132 treatment effectively protected the kidneys of rats against the complications of DM.

In summary, we showed that MG132 is a proteasome inhibitor that can effectively provide renoprotection in DN rats via inhibition of the PI3K/Akt pathway-related inflammatory response. Although the precise mechanism should be explored in future studies, and one must be cautious in applying animal models to human disease, these studies provide a theoretical basis for further study of the clinical prevention and treatment of DN.
